# Waist circumference, waist-to-hip ratio and waist-to-height ratio reference percentiles for abdominal obesity among Greek adolescents

**DOI:** 10.1186/s12887-015-0366-z

**Published:** 2015-05-04

**Authors:** Flora Bacopoulou, Vasiliki Efthymiou, Georgios Landis, Anastasios Rentoumis, George P Chrousos

**Affiliations:** Center for Adolescent Medicine and UNESCO Chair in Adolescent Health Care, First Department of Pediatrics, University of Athens Medical School, Aghia Sophia Children’s Hospital, 3 Thivon Street, Athens, 115 27 Greece

**Keywords:** Percentiles, Waist circumference, Waist-hip ratio, Waist-height ratio, Abdominal obesity, Greece, Adolescents

## Abstract

**Background:**

Indices predictive of adolescent central obesity include waist circumference (WC), waist-to-hip ratio (WHR) and waist-to-height ratio (WHtR). Such reference data are lacking for Greek adolescents. The aim of this study was to develop age- and gender-specific WC, WHR and WHtR smoothed reference percentiles for abdominal obesity among Greek adolescents aged 12–17 years, to investigate possible obesity cut-offs of WHR and WHtR and to compare WC percentiles to other adolescent populations.

**Methods:**

A representative sample of 1610 high school adolescents (42.2% boys, 57.8% girls; mean age ± sd 14.4 ± 1.72 years) participated in this cross-sectional study in Attica, Greece, in 2013. Weight, height, body mass index (BMI), WC, hip circumference (HC), WHR and WHtR were measured and percentiles were calculated using the LMS method. The relation between WHR, WHtR and general obesity, as defined by the International Obesity Task Force, was investigated with receiver operating characteristic (ROC) analysis. The discriminating power of WHR and WHtR was expressed as area under the curve (AUC). Greek adolescents’ WC measurements at the 50th and 90th percentile were compared with their counterparts’ smoothed percentiles from Norway, Turkey, Poland, South India, Germany and Kuwait.

**Results:**

Boys had significantly higher mean in all measures than girls, except for BMI where there was no statistical difference in terms of gender. BMI, WC and HC showed an increasing trend with age. WC leveled off in both genders at the age of 17 years. WHR and WHtR showed a continuous decrease with advancing age. WHtR was a better predictor for general obesity in both boys and girls (AUC 95% CI 0.945-0.992) than the WHR (AUC 95% CI 0.758-0.870); the WHtR cut-off of 0.5 had sensitivity 91% and specificity 95% for both genders and all age groups combined. International comparisons showed that Greek adolescents had relatively high levels of abdominal obesity in early-middle adolescence but this did not persist at the age of 17 years.

**Conclusions:**

These reference percentile curves could be used provisionally for early detection of abdominal obesity in Greek adolescents aged 12–17 years; WHtR of 0.5 could also be used as a threshold for obesity in this age group.

## Background

The worldwide prevalence of overweight (OW) and obesity (OB) in children and adolescents has reached worrying levels. School-based studies using standardized Greek or international cut-off points, have shown high prevalence of adolescent OW and OB over the two past decades in Greece. A nationwide study carried out during 1997–1998 in a representative sample of adolescents 11–16 years of age reported an overall prevalence of OW and OB 15.3% and 1.8% respectively, according to the International Obesity Task Force (IOTF) criteria [1,2]. In a large-scale epidemiological survey throughout all parts of Greece in 2003, the overall prevalences of OW and OB for adolescents aged 13–19 years were 23.3% and 6.1% in boys vs. 14% and 2.7% in girls, respectively [3]. A regional study in the second largest Greek city of Thessaloniki reported a prevalence of OW and OB of 19% and 2.6% for the ages 11–17 years [4]. For the same city, another study showed a prevalence of OW and OB of 23.8% and 4.8% according to IOTF cut-off points, for the ages 12–18 years [5].

The above findings were based on body mass index (BMI), which gives no indication of distribution of body fat. In adolescents, as in adults, central or abdominal fat increases the risk for metabolic (dyslipidemia and insulin resistance) and cardiovascular complications [6,7]. Indices predictive of adolescent central obesity include waist circumference (WC), waist-to-hip ratio (WHR) and waist-to-height ratio (WHtR). WC is a highly sensitive and specific measure of upper body fat in young people and thus it is valuable for identifying overweight and obese adolescents at risk of developing metabolic complications. The same applies for risk factors of cardiovascular disease in children and adolescents, in whom WC and WHtR are better predictors than BMI [8].

Previous literature suggests the use of pre-specified cut-off points for defining central obesity; WHtR ≥0.5 [9], WHR > 0.90 in men and > 0.85 in women, in order to standardize comparisons within and between populations [10]. However such cut-off points may not be suitable for all adolescents, as the sensitivity and specificity change with age end ethnicity. Moreover, developing ethnic-specific cut-offs requires a demonstration of differential predictive validity. Only few specific cut-offs or even reference percentiles for WC, WHR, WHtR are available for adolescents in some countries [11-17] and differ from one country to another, due to genetic and environmental factors. Such reference data are lacking for Greek adolescents, thus comprehensive reference values for WC, WHR, WHtR are needed in order to establish an ethnic accepted age- and gender-specific definition of central obesity for adolescents in Greece.

The aim of this study was to develop age- and gender-specific WC, WHR and WHtR smoothed reference percentiles for abdominal obesity among Greek adolescents 12–17 years of age, to compare them to worldwide curves generated for other adolescent populations and to investigate possible WHR and WHtR cut-offs for detecting general obesity as defined by the IOTF.

## Methods

### Subjects

Cross-sectional anthropometric data were obtained from adolescents aged 12–17 years, as part of a “program for health promotion, prevention and screening for characteristics of metabolic syndrome in adolescents attending high schools in three municipalities (Palaio Faliro, Aghios Dimitrios, Alimos) in the Attica region in Greece, with the use of portable telemedicine”. The program was funded by the European Union and conducted from September to November 2013. The study was in agreement with the Helsinki Declaration. Ethical approval was obtained from the Uniform Administrative Sector of Primary and Secondary Education of the Greek Ministry of Education and Religious Affairs (reference number 86758/Γ2).

**Figure 1:**
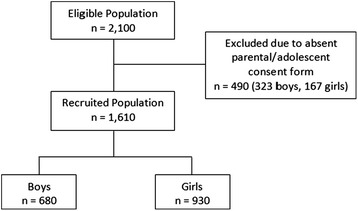
Eligible and finally recruited adolescent population.

Attica includes the capital and surrounding urban areas; it is the largest and most populated region in Greece. The inhabitants are quite similar to the general Greek population as the urban population represents 60% of the country population and people from anywhere in Greece have been immigrating to Athens in the last 70 years. A total of 23 public high schools were randomly selected and the adolescents attending the schools were informed about the study. All adolescents who provided a consent form signed by them and their parents or guardians, participated in the study. A total of 2,100 adolescents were initially contacted, however, 490 eligible adolescents (323 boys, 167 girls) were excluded from the measurements as they did not provide consent forms signed by them and their parents or guardians. Finally, a total of 1610 adolescents (680 males and 930 females) were included in the study (Figure 1). As far as possible, the sample was representative of the Attica population of adolescent girls and boys. The adolescent population aged 12 to 17years was estimated at 212,189 adolescents in the middle of 2011. The population sample of 1610 adolescents satisfied a margin error of 3.2% at a 99% confidence interval.

### Measurements

Sampled schools were visited on pre-arranged dates, early in the morning, by a team of pediatricians and nurses, appropriately trained to perform anthropometry by the principal investigator and an exercise physiologist.

Participants were instructed to fast overnight before screening. The study protocol included the anthropometric parameters body weight (BW), height (Ht), WC and hip circumference (HC), with adolescents being bare-foot and in minimal clothing. Adolescents’ weight was measured with electronic portable scales (Fora w100b, Switzerland) connected to suitable telemedicine software (vida24, Vidavo Health Telematics). Height was measured by portable stadiometers with the participants’ feet placed together with heels, buttocks and shoulder blades against the stick and head positioned in the Frankfurt horizontal plane. Waist and hip circumferences were measured twice using inextensible anthropometric tape with the adolescents standing erect and relaxed with arms at the sides and feet positioned close together. Waist circumference was measured midway between the lowest border of rib cage and the upper border of iliac crest, at the end of normal expiration [18]. HC was measured at the widest part of the hip at the level of the greater trochanter. For all measurements the tape was positioned at a level parallel to the floor. All measurements were in centimeters (cm) to the nearest 0.1cm. For each participant, measurements were registered in the software database.

### Statistical analysis

Mean values were calculated for BW, Ht, BMI, WC, HC, WHR and WHtR. BMI was calculated as the ratio of body weight to the square of height (kg/m^2^), WHR was calculated as the ratio of waist to hip circumference and WHtR as the ratio of waist circumference to height. Smoothed age- and gender-specific table and graph percentiles were constructed for BMI, WC, WHR and WHtR by the LMS method. The 3rd, 10th, 25th, 50th, 75th, 90th and 97th percentiles were calculated to be in agreement with the official Greek BMI curves for adolescents [19].

To evaluate the representativeness of the study data, we examined if the study participants’ BMI had equal means with the BMI derived from the Greek official growth curves for age-matched adolescents. Using Student t-test, we found that there was no statistical difference between these two populations.

Statistical analyses were carried out using the SPSS software 21 version for Windows. Smoothed age and gender-specific curves for all percentiles were constructed with the use of software package LMS Chart Maker Pro, version 2.54. The LMS method assumes that the data can be normalized by using power transformation. The percentile curves were the results of smoothing the age specific curves: L for skewness, M for median and S for coefficient of variation [20,21].

The relation between WHR, WHtR and general obesity as defined by the IOTF was investigated with nonparametric receiver operating characteristic (ROC) analysis. The discriminating power of the WHR and the WHtR was expressed as area under the curve (AUC) ± std. error or 95% confidence intervals (CI).

## Results

The study included a sample of 1610 high school adolescents aged 12 to 17 years. The male to female ratio was 0.73 with 42.2% boys and 57.8% girls. The mean age (±sd) was 14.4 (±1.72) years. Descriptive statistics for BW, Ht, BMI, WC, HC, WHR and WHtR, by age group for boys and girls are presented in Table 1. Boys had a significantly higher mean in all measures than girls, except for BMI where there was no statistical difference in terms of gender. BMI, WC and HC showed an increasing trend with age. WC leveled off in both genders at the age of 17years. WHR and WHtR showed a continuous decrease with advancing age.

**Table 1:** **Mean values (±sd) for body weight (BW), height (Ht), body mass index (BMI), waist circumference (WC), hip circumference (HC), waist-to-hip ratio (WHR) and waist-to-height ratio (WHtR) for Greek adolescents aged 12–17 years**

**Age (years)**	**Number**	**BW (kg)**	**Ht (cm)**	**ΒΜΙ** **(kg/m** ^**2**^ **)**	**WC (cm)**	**HC (cm)**	**WHR**	**WHtR**
**Boys**								
12	127	52.0 ± 11.6	157.0 ± 7.8	21.0 ± 3.8	71.4 ± 9.4	88.9 ± 8.2	0.80 ± 0.06	0.45 ± 0.06
13	137	57.9 ± 13.1	164.1 ± 7.1	21.4 ± 4.1	73.5 ± 11.0	91.2 ± 9.3	0.80 ± 0.07	0.45 ± 0.06
14	102	60.2 ± 10.8	168.3 ± 7.4	21.1 ± 3.1	73.5 ± 8.2	92.8 ± 7.3	0.79 ± 0.05	0.44 ± 0.05
15	113	66.5 ± 12.1	174.1 ± 7.1	21.9 ± 3.6	74.0 ± 8.4	95.6 ± 8.2	0.77 ± 0.05	0.43 ± 0.05
16	122	70.0 ± 13.0	176.4 ± 6.5	22.4 ± 3.7	76.1 ± 9.0	97.6 ± 8.0	0.78 ± 0.06	0.43 ± 0.05
17	79	71.2 ± 11.7	177.0 ± 6.8	22.6 ± 3.4	74.8 ± 7.8	96.9 ± 7.6	0.77 ± 0.06	0.42 ± 0.04
Total	680	62.3 ± 13.9	168.8 ± 10.2	21.7 ± 3.7	73.8 ± 9.3	93.6 ± 8.8	0.79 ± 0.06	0.44 ± 0.05
**Girls**								
12	159	49.8 ± 10.9	156.5 ± 7.0	20.2 ± 3.6	68.1 ± 9.0	89.3 ± 8.2	0.76 ± 0.06	0.43 ± 0.05
13	156	54.3 ± 9.7	160.4 ± 5.9	21.0 ± 3.4	68.8 ± 8.7	93.6 ± 9.6	0.74 ± 0.05	0.43 ± 0.05
14	117	56.6 ± 10.6	162.0 ± 5.9	21.5 ± 3.7	69.8 ± 9.0	94.4 ± 8.3	0.74 ± 0.08	0.43 ± 0.06
15	180	57.3 ± 9.6	162.5 ± 6.1	21.6 ± 3.0	68.4 ± 6.9	96.0 ± 6.2	0.71 ± 0.04	0.42 ± 0.04
16	180	58.8 ± 9.5	163.5 ± 6.1	21.9 ± 3.1	68.9 ± 7.2	97.1 ± 8.5	0.71 ± 0.04	0.42 ± 0.04
17	138	59.4 ± 9.8	163.9 ± 6.0	22.1 ± 3.3	68.7 ± 7.2	97.2 ± 8.3	0.71 ± 0.04	0.42 ± 0.05
Total	930	56.0 ± 10.5	161.5 ± 6.7	21.4 ± 3.4	68.7 ± 7.9	94.6 ± 8.6	0.73 ± 0.06	0.43 ± 0.05

**Figure 2:**
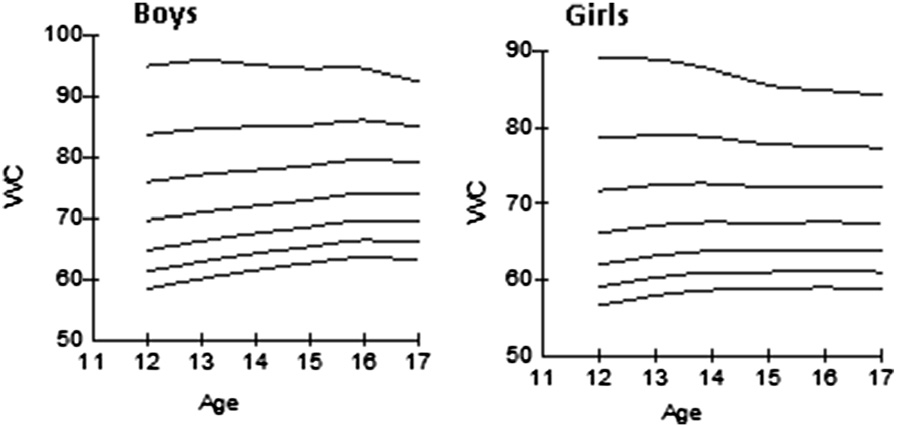
Smoothed waist circumference (WC in cm) percentile curves for Greek adolescents.

**Figure 3:**
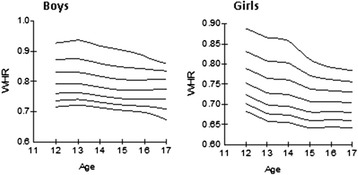
Smoothed waist-to-hip ratio (WHR) percentile curves for Greek adolescents.

**Figure 4:**
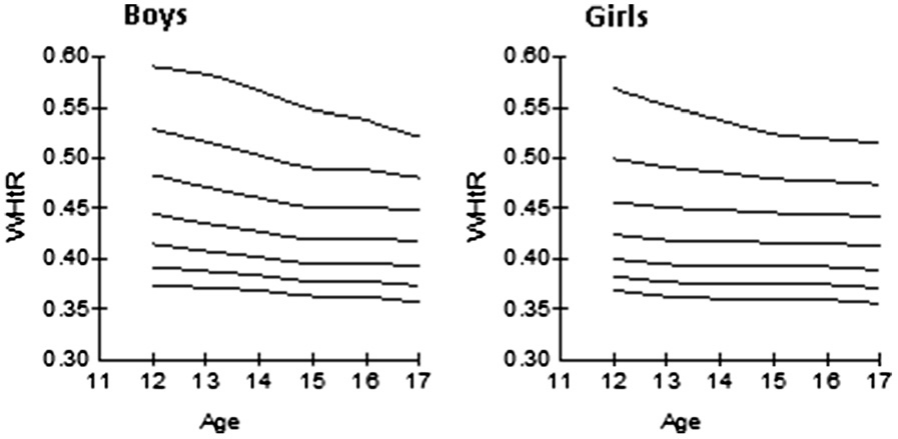
Smoothed waist-to-height ratio (WHtR) percentile curves for Greek adolescents.

The following tables and curves demonstrate the smoothed gender- and age-specific percentile values at the 3rd, 10th, 25th, 50th, 75th, 90th and 97th percentiles, which were developed and smoothed by the LMS method; for WC [Table 2, Figure 2], for WHR [Table 2, Figure 3] and for WHtR [Table 2, Figure 4].

**Table 2:** **Age- and gender-specific smoothed body mass index (BMI), waist circumference (WC), waist-to-hip ratio (WHR) and waist-to-height ratio (WHtR) percentiles for Greek adolescents aged 12–17 years**

	**Age (years)**	**Percentiles**						
		**3rd**	**10th**	**25th**	**50th**	**75th**	**90th**	**97th**
**Boys**								
**BMI** (kg/m^2^)	12	15.7	16.8	18.3	20.3	22.8	25.9	30.0
	13	16.2	17.3	18.6	20.5	22.9	25.9	30.3
	14	16.6	17.7	18.9	20.7	23.0	25.8	29.9
	15	17.2	18.2	19.5	21.2	23.4	26.2	30.3
	16	17.6	18.6	19.9	21.6	23.9	26.7	30.6
	17	18.0	19.0	20.3	22.0	24.3	27.0	30.7
**WC** (cm)	12	58.4	61.3	64.8	69.7	76.0	83.8	95.0
	13	60.1	62.9	66.3	71.0	77.2	84.8	95.9
	14	61.6	64.3	67.6	72.1	77.9	85.0	95.2
	15	62.7	65.4	68.6	73.0	78.6	85.3	94.6
	16	63.7	66.5	69.8	74.2	79.7	86.1	94.6
	17	63.3	66.1	69.5	73.8	79.2	85.1	92.5
**WHR**	12	0.72	0.74	0.76	0.79	0.83	0.87	0.93
	13	0.72	0.74	0.76	0.79	0.83	0.88	0.94
	14	0.71	0.73	0.75	0.78	0.82	0.86	0.92
	15	0.70	0.72	0.74	0.77	0.81	0.85	0.90
	16	0.70	0.72	0.74	0.77	0.81	0.84	0.88
	17	0.67	0.71	0.74	0.78	0.81	0.83	0.86
**WHtR**	12	0.37	0.39	0.41	0.44	0.48	0.53	0.59
	13	0.37	0.39	0.41	0.43	0.47	0.52	0.58
	14	0.37	0.38	0.40	0.43	0.46	0.50	0.57
	15	0.36	0.38	0.39	0.42	0.45	0.49	0.55
	16	0.36	0.38	0.40	0.42	0.45	0.49	0.54
	17	0.36	0.37	0.39	0.42	0.45	0.48	0.52
**Girls**								
**BMI** (kg/m^2^)	12	15.2	16.4	17.8	19.6	22.0	24.8	28.5
	13	16.0	17.2	18.6	20.5	22.8	25.5	28.9
	14	16.4	17.6	19.0	20.9	23.2	25.7	28.9
	15	16.9	18.1	19.4	21.2	23.4	25.9	28.9
	16	17.3	18.4	19.7	21.5	23.6	26.0	29.1
	17	17.4	18.5	19.8	21.5	23.6	26.1	29.4
**WC** (cm)	12	56.7	59.1	62.1	66.2	71.7	78.6	89.2
	13	58.0	60.3	63.2	67.2	72.4	79.0	88.9
	14	58.7	61.0	63.8	67.6	72.6	78.7	87.6
	15	58.8	61.1	63.8	67.5	72.2	77.8	85.6
	16	59.0	61.3	64.0	67.7	72.3	77.6	84.9
	17	58.7	61.0	63.8	67.5	72.0	77.3	84.3
**WHR**	12	0.68	0.70	0.72	0.75	0.79	0.83	0.89
	13	0.66	0.68	0.70	0.73	0.76	0.81	0.87
	14	0.65	0.67	0.70	0.72	0.76	0.80	0.86
	15	0.64	0.66	0.68	0.71	0.74	0.77	0.81
	16	0.64	0.66	0.68	0.71	0.73	0.76	0.79
	17	0.64	0.66	0.68	0.70	0.73	0.76	0.78
**WHtR**	12	0.37	0.38	0.40	0.42	0.46	0.50	0.57
	13	0.36	0.38	0.39	0.42	0.45	0.49	0.55
	14	0.36	0.38	0.39	0.42	0.45	0.49	0.54
	15	0.36	0.37	0.39	0.42	0.45	0.48	0.52
	16	0.36	0.37	0.39	0.42	0.44	0.48	0.52
	17	0.36	0.37	0.39	0.41	0.44	0.47	0.52

**Figure 5:**
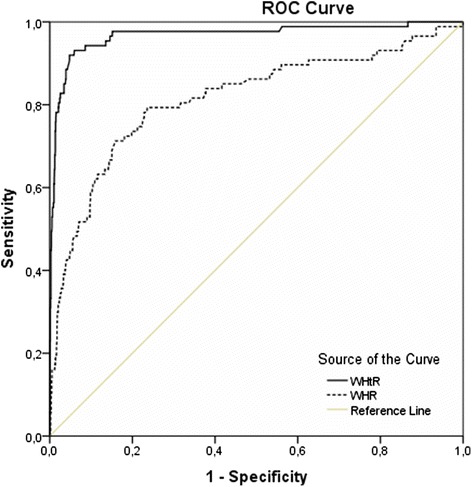
Receiver operating characteristic (ROC) curve for prediction of general obesity from waist-to-height ratio (WHtR) and waist-to-hip ratio (WHR) in both genders.

ROC analysis showed that WHtR had higher discriminating power to detect IOTF obesity than WHR [Figure 5]. For obesity, the WHtR cut-off of 0.5 had a sensitivity of 91% and a specificity of 95% for both genders (AUC 0.969 ± 0.018 for boys, 0.968 ± 0.015 for girls) and all age groups combined (AUC 0.968 ± 0.012). The cut-off for WHR > 0.90 in boys had a sensitivity of 22% and a specificity of 97% (AUC 0.866 ± 0.028) whereas the WHR > 0.85 cut-off in girls had a sensitivity of 24% and a specificity of 99% (AUC 0.772 ± 0.047). WHtR was a better predictor of general obesity than the WHR in both boys and girls.

### Comparisons

**Figure 6:**
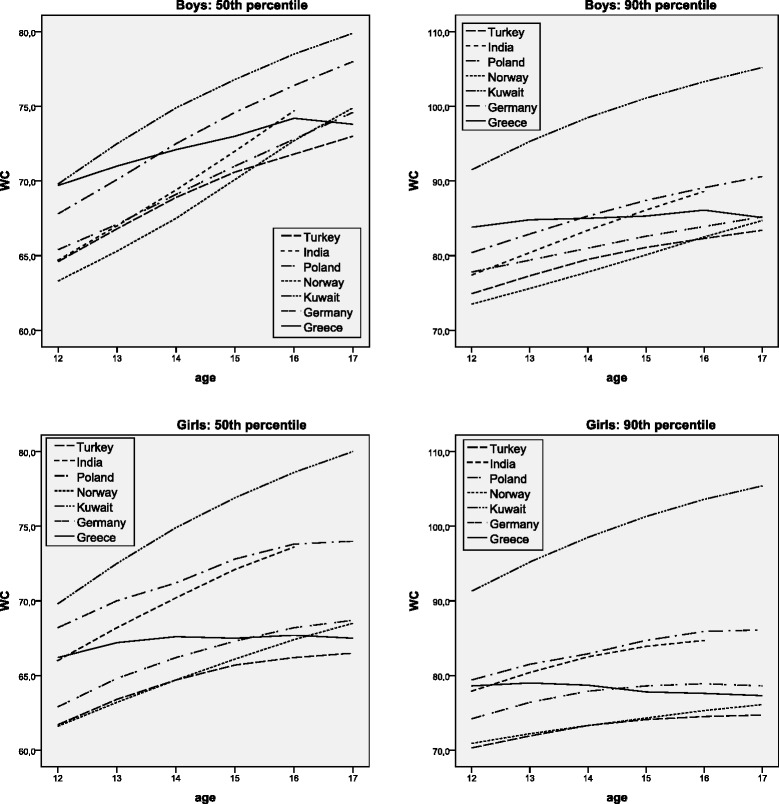
Comparison of the 50th and 90th percentile LMS smoothed waist circumference (WC in cm) reference curves for boys and girls from seven countries.

We compared our WC reference curves with those of other countries in an attempt to evaluate ethnic differences in adolescent abdominal obesity. Because of different methods that have been used in published national reference values for adolescents, caution is needed when comparing WC percentile reference data between studies. Therefore, comparison was limited among studies that used the LMS method. To use contemporary data, only surveys conducted from the year 2000 onwards were included. The results of the Greek adolescents’ WC measurements were compared with those from countries of diverse geographical location and ethnicity: Norway, Turkey, Poland, South India, Germany and Kuwait [17,22-26]. Percentile curves for these countries were also based on cross-sectional data obtained from adolescents in the school setting. For the adolescents aged 12–17 years the reported mean BMIs ranged from 20.4 to 24.2 for boys and from 20.3 to 23.9 for girls [23,25,26]. Our study participants had mean BMI values of 21.7 for boys and 21.4 for girls (Table 1). Data about WC is of special interest at the 50th percentile (WC_50_) because they reflect the majority as a median point and at the 90th percentile (WC_90_) because they reflect the pathological point of abdominal obesity. Comparison of WC_50_ (median) and WC_90_ (abdominal obesity cut-off) for Greek adolescents with their counterparts’ smoothed percentiles developed over the past 15years in six countries, are presented in Figure 6.

Our findings showed that Kuwaiti adolescents had higher WC and Turkish adolescents had lower WC than Greeks in all ages for both genders. Greek adolescents had higher WC values compared to South Indian, Polish and Norwegian adolescents at all ages except at the age of 17 years, where WC dropped to lower levels. Greek adolescents in general had lower WC than Germans except at the ages of 12–13 years, where Greek boys did worse. Inter-country comparisons were also performed for adolescents’ WC_50_ and WC_90_ at the mean age of 14years as shown in Table 3. Results showed that 50th and 90th WC percentile curves for Greek adolescents aged 14years were higher for girls and boys as compared to those for Norwegian, Turkish, and Polish adolescents but lower than German and Kuwaiti girls and boys. WC_50_ and WC_90_ were higher for Greek boys and lower for girls compared with their counterparts from South India.

**Table 3:** **Comparison of WC**_**50**_ **(median) and WC**_**90**_ **(cut-off) for waist circumference (in cm) among 14-year old boys and girls from different countries**

**Boys**			**Girls**		
	**WC**_**50**_	**WC**_**90**_		**WC**_**50**_	**WC**_**90**_
Norway	67.5	77.8	Norway	64.7	73.3
Turkey	68.9	79.5	Turkey	64.7	73.3
Poland	69.1	81.0	Poland	66.2	77.9
South India	69.4	83.4	South India	70.2	82.5
Greece	72.1	85.0	Greece	67.6	78.7
Germany	72.5	85.3	Germany	71.2	82.9
Kuwait	74.9	98.5	Kuwait	74.9	98.5

## Discussion

This study presents the first age- and gender-specific smoothed percentiles for WC, WHR and WHtR for Greek adolescents 12 to 17 years of age. Percentile data were developed from a representative sample of 1610 Greek high school adolescents. These curves represent the first attempt to develop smoothed percentile curves for those indices and to suggest Greek cut offs for defining abdominal obesity in this population.

Consistent with previous literature in adolescents [11,13,17,22-29], WC showed an increasing trend with age among both girls and boys. This is an expected finding during puberty, as it represents a critical period for body fat development and distribution [30]. At the upper end of the age range, WC leveled off in both genders. Boys had higher WC, WHR and WHtR values than girls at all ages. This probably reflects gender-specific influences on waist circumference and can be explained by the fact that in boys central fat distribution is more predominant than in girls. Adipose tissue is distributed mainly in the upper body (nape of neck, shoulders, epigastrium) in boys, whereas in girls peripheral fat accumulation in the lower body predominates.

Data about WC percentiles might be of special interest as it remains the most widely accepted method and the simplest clinical measure of pubertal central obesity; it is noninvasive and easy to obtain. For adults, a cut-off for predicting the risk for metabolic syndrome exists, however, adolescents require separate cut-offs of sex-specific WC norms relative to age because of the normal increase in WC throughout puberty. The 90th percentile for WC is commonly suggested [31-33] as a cut-off percentile at and above which the risk for metabolic syndrome in adolescence increases substantially. As WC cut off values vary according to ethnicity and such data are lacking for Greek adolescents, we aimed to establish the 90th percentile for WC in an effort to provide population specific reference values for this age group. Provided that a specific WC cut-off exists, measurement of this anthropometric indicator will be a useful screening tool for cardiovascular and metabolic disease risk in adolescents in primary care practice in Greece.

In young children, WC is reported to be a better estimate of body fat percentage when adjusting for gender and age, thus pointing to the importance of examining age-range specific subgroups [34]. When all of our study adolescents were combined, mean BMI increased with age and 11.1% were found to have obese waist circumference (based on their 90th percentile). The percentage of adolescents who exceeded the 90th percentile reached a peak at the age of 13 years (averaging about 14%) and showed a decrease at the age of 15 years (averaging about 8.6%). Thus, the age group of 13 years appeared to be at increased risk for central obesity but this risk seemed to decrease after the age of attainment of peak height velocity.

International comparisons showed that Greek adolescents have relatively high levels of abdominal obesity in early and middle adolescence and this seems to reverse at the age of 17 years. Unhealthy eating habits and other lifestyle patterns are related to obesity in Greek adolescents [35,36]. The relative decline in abdominal obesity at the age of 17 years could be explained by the teenagers’ personal (exploration of identity) and social motivation (peer pressure, sexual experimentation) during transition to mid-adolescence, as body mass seems to affect body dissatisfaction and self-esteem in both girls and boys [37]. The major differences of the percentile curves among various countries confirm the evidence of ethnic differences in abdominal obesity and the ongoing need for providing population specific WC reference curves for adolescents.

WHR and WHtR exhibited an age-dependent decrease for both genders. The cut-off of WHR > 0.90 corresponded at the 97th WHR percentile for adolescent boys whereas the cut-off of > 0.85 corresponded at the 93rd-99th percentiles in girls according to age. These WHR cut-offs used in adults were not suitable to be used as a threshold for general obesity in our adolescents because of a low sensitivity demonstrated by ROC analysis. Previous literature suggests the cut-off of WHtR ≥ 0.5 as a useful predictor of central obesity in representative samples of UK, Norwegian and German adolescents [9,23,26]. This finding was verified in our representative sample of Greek adolescents. We observed that this cut-off corresponded to the 85th-90th WHtR percentiles at all ages for both girls and boys. In addition, the receiver operating curves (ROC) demonstrated that WHtR was a better predictor for general obesity in both boys and girls (AUC 95% CI 0.945-0.992) than the WHR, which demonstrated a smaller AUC (95% CI 0.758-0.870) to detect obesity in both genders according to the IOTF criteria. We conclude that WHtR of 0.5 could also be used as a threshold for obesity in Greek adolescents 12–17 years of age.

Our study had several strengths such as the fact that similar studies have not been previously performed in Greek adolescents. Anthropometric data were collected by appropriately trained health professionals that used the same anatomic sites of measurements. Furthermore, results are likely to be representative of today’s adolescents because BMI data were collected during a recent specific period of time and were similar to contemporary Greek official adolescent BMI data.

Limitations include the lack of information of the effect of pubertal status on the anthropometric indices, as well as the cross-sectional design of the study based on data collection in 2013 with a high prevalence of overweight/obesity. The prevalence of pediatric and adolescent overweight/obesity in Greece has risen to more than 30% over the past 5 years [38], however the lack of earlier analytic data did not allow accurate comparisons. Although our reference lines are based on contemporary data that are likely to be representative of the current situation in Greece, validation of these percentile curves against equivalent longitudinal data are warranted in future studies. Another limitation was the lower participating rate of boys vs. girls. The higher percentage of boys’ missing consent forms was mainly attributed to their negligence to inform their parents. The fact that more girls had taken care for the consent forms to be completed in time, could be partially attributed to their higher interest in body measurement, size and shape as they are more sensitive to their changing physical appearance, than boys [39].

## Conclusions

In conclusion, this is the first comprehensive study which determines smoothed age- and sex-specific WC, WHR and WHtR percentiles in the Greek population for adolescents aged 12 to 17 years. We propose that these percentiles could be used provisionally in clinical practice for early detection of abdominal obesity in Greek adolescents. WHtR could be used as an additional or alternative screening tool for general obesity in this age group.

## Abbreviations

OW: Overweight
OB: Obesity
IOTF: International Obesity Task Force
BMI: Body mass index
WC: Waist circumference
WHR: Waist-to-hip ratio
WHtR: Waist-to-height ratio
BW: Body weight
Ht: Height
HC: Hip circumference
ROC: Receiver operating characteristic
AUC: Area under the curve
WC_50_: Waist circumference at the 50th percentile
WC_90_: Waist circumference at the 90th percentile
